# Distinct BTK inhibitors differentially induce apoptosis but similarly suppress chemotaxis and lipid accumulation in mantle cell lymphoma

**DOI:** 10.1186/s12885-021-08475-3

**Published:** 2021-06-26

**Authors:** Zhuojun Liu, Jia Liu, Tianming Zhang, Lin Li, Shuo Zhang, Hao Jia, Yuanshi Xia, Mingxia Shi, Jing Zhang, Shuhua Yue, Xiaofang Chen, Jian Yu

**Affiliations:** 1 Interdisciplinary Institute of Cancer Diagnosis and Treatment, Beijing Advanced Innovation Center for Biomedical Engineering, Beihang University, Beihang University, Beijing, 100083 China; 2 School of Biological Science and Medical Engineering, Beihang University, Beijing, 100083 China; 3 Department of Hematology, the First Affiliated Hospital of Kunming Medical University, Kunming, China

**Keywords:** Mantle cell lymphoma, Ibrutinib, Acalabrutinib, Zanubrutinib, Apoptosis, Chemotaxis, Lipid droplet accumulation

## Abstract

**Background:**

The more selective second-generation BTK inhibitors (BTKi) Acalabrutinib and Zanubrutinib and the first-generation BTKi Ibrutinib are highlighted by their clinical effectiveness in mantle cell lymphoma (MCL), however, similarities and differences of their biological and molecular effects on anti-survival of MCL cells induced by these BTKi with distinct binding selectivity against BTK remain largely unknown.

**Methods:**

AlamarBlue assays were performed to define cytotoxicity of BTKi against MCL cells, Jeko-1 and Mino. Cleaved PARP and caspase-3 levels were examined by immunoblot analysis to study BTKi-induced apoptotic effects. Biological effects of BTKi on MCL-cell chemotaxis and lipid droplet (LD) accumulation were examined in Jeko-1, Mino and primary MCL cells via Transwell and Stimulated Raman scattering imaging analysis respectively. Enzyme-linked immunoassays were used to determine CCL3 and CCL4 levels in MCL-cell culture supernatants. RNA-seq analyses identified BTKi targets which were validated by quantitative RT-PCR (qRT-PCR) and immunoblot analysis.

**Results:**

Acalabrutinib and Zanubrutinib induced moderate apoptosis in Ibrutinib high-sensitive JeKo-1 cells and Ibrutinib low-sensitive Mino cells, which was accompanied by cleaved PARP and caspase-3. Such effects might be caused by the stronger ability of Ibrutinib to upregulate the expression of pro-apoptotic genes, such as *HRK, GADD45A*, and *ATM*, in JeKo-1 cells than in Mino cells, and the expression of such apoptotic genes was slightly changed by Acalabrutinib and Zanubrutinib in both JeKo-1 and Mino cells. Further, Acalabrutinib, Zanubrutinib and Ibrutinib reduced MCL-cell chemotaxis with similar efficiency, due to their similar abilities to downmodulate chemokines, such as CCL3 and CCL4. Also, these three BTKi similarly suppressed MCL-cell LD accumulation via downregulating lipogenic factors, DGAT2, SCD, ENPP2 and ACACA without significant differences.

**Conclusion:**

BTKi demonstrated differential capacities to induce MCL-cell apoptosis due to their distinct capabilities to regulate the expression of apoptosis-related genes, and similar biological and molecular inhibitory effects on MCL-cell chemotaxis and LD accumulation.

**Supplementary Information:**

The online version contains supplementary material available at 10.1186/s12885-021-08475-3.

## Background

Mantle cell lymphoma (MCL) is defined as a highly aggressive mature B-cell neoplasm in the WHO classification [1]. Among the pathways supporting MCL-cell survival, B-cell receptor (BCR) signaling plays a prominent role [2]. Bruton tyrosine kinase (BTK), a key component of BCR signaling, is an attractive target for the treatment of B-cell malignancies, including MCL [3]. Ibrutinib is the first-class BTK inhibitor binding to Cys-481 residue in the ATP binding domain of BTK irreversibly, and thereby impairing MCL-cell survival via inhibiting BCR signaling. Ibrutinib could potently induce apoptosis via inhibiting BCR signaling activated canonical NF-κB signaling in MCL [4], or altering the expression of anti-apoptotic gene, MCL-1 [5]. Furthermore, blockage of BCR signaling could reduce MCL-cell homing into microenvironment in lymphoid organs or bone marrow and thereby inhibiting MCL-cell survival, which was considered as the predominant action of BTKi [6]. The production of chemokines, CCL3 and CCL4, is increased upon BCR activation in MCL [6], chronic lymphocytic leukemia (CLL) [7] and diffuse large B cell lymphoma (DLBCL) [8], and such effects can be impaired by Ibrutinib treatment, which also significantly decreases chemokine receptor CXCR4 expression in MCL [6] and CLL [9]. Recently, lipid metabolism in cancer cells has received increased interest for therapeutic interventions and some tumor cells have prominent lipid droplets, which may associated with the enhanced cell viability, aggressiveness and chemotherapy resistance [10]. It has been reported that BCR signaling activation could increase the level of lipoprotein lipase (LPL), a protein essential for fatty acid metabolism providing cells with energy and survival advantage, which can be impaired by Ibrutinib treatment via reducing LPL level in CLL [11].

Acalabrutinib and Zanubrutinib are the second-generation covalent BTK inhibitors binding to Cys-481 residue of BTK irreversibly with more selectivity [12, 13]. Similar with Ibrutinib, Acalabrutinib treatment inhibits the migratory capacity of CLL cells mediated by tissue-homing chemokines, CCL3 and CCL4 [14, 15], and induces CLL-cell apoptosis accompanied by cleavage of PARP and caspase-3 via inhibiting the activation of ERK and AKT [16]. Moreover, Acalabrutinib treatment is associated with inhibition of fatty acid metabolism induced by fatty acid synthase (FASN) downregulation, which may trigger significant apoptosis of MCL [17] or CLL cells [14]. Zanubrutinib could inhibit homing of CLL cells through downregulating homing receptors such as CXCR5 [18], and effectively disrupt AKT/mTOR signaling and NF-κB function, leading to MCL-cell apoptosis [19]. Furthermore, Zanubrutinib downmodulates two metabolic enzymes involved in fatty acid synthesis, FASN and Acetyl-CoA carboxylase 1 (ACC1), which might potentially impair MCL-cell survival via inhibiting lipid metabolism [19].

Although Ibrutinib, Acalabrutinib and Zanubrutinib have similar biologic effects and comparable clinical responses, molecular mechanisms underlying their anti-MCL activities might be differential due to their distinct binding selectivity against BTK [12, 13]. In this study, we performed transcriptome-wide RNA sequencing analyses to identify the target genes of Ibrutinib, Acalabrutinib or Zanubrutinib in MCL cells, by which addressed molecular mechanisms underlying the induction of MCL-cell apoptosis and inhibition of MCL-cell chemotaxis and LD accumulation induced by these three BTKi.

## Methods

### Cell culture and reagents

MCL cell lines, JeKo-1 and Mino, or HS-5 human stromal cell line were obtained from ATCC, and cultured in RPMI1640 or DMEM medium (Hyclone, Waltham, MA) respectively, containing 10% fetal bovine serum (FBS; GIBCO, Carlsbad, CA), 2 mM L-glutamine, 100 U/ml penicillin and 100 μg/ml streptomycin. The cells with passage number 6 were used for the experiments. Untreated specimens of bone marrow aspirates were obtained from patients with MCL after they provided informed consents, in compliance with the Declaration of Helsinki, and approved by the Institutional Review Board at both Beihang University and the First Affiliated Hospital of Kunming Medical University. Mononuclear cells were separated by Ficoll-Hypaque density centrifugation, and primary MCL cells were isolated by the use of anti-CD19 microbeads (#130–050-301, Miltenyi Biotec), and total of 3 cases were tested in this research. Primary MCL cell cultures were maintained in RPMI 1640 medium containing 10% FBS, 2 mM L-glutamine, 100 U/ml penicillin and 100 μg/ml streptomycin. All the cells were maintained at 37 °C in a humidified atmosphere of 5% CO_2_. Ibrutinib (HY-10997), Acalabrutinib (HY-17600) and Zanubrutinib (HY-101474) were purchased from MedChem Express (Shanghai, China).

### Chemotaxis assay

MCL cells were serum-starved for 12 h and then treated with or without each BTKi (2 μM) for 6 h. A total of 5 × 10^5^ cells were seeded in the upper compartment of Transwell culture polycarbonate insert with 6.5-mm diameter and 5 μm of pore size (Corning). Cells were incubated for 8 h in serum free medium at 37 °C and 5% CO_2_ and the migration toward HS-5 conditioned medium was analyzed by flow cytometry (FACScan) for 1 min under constant flow rate. The percentage of migrating cells was calculated as the number of migrated cells divided by total number of input cells.

### RNA-seq analysis

BTKi treated (2 μM) and untreated cells were harvested in Trizol reagent (Invitrogen) and total RNA extraction operation was the same with that of qRT-PCR section. Sequence libraries were generated and sequenced by CapitalBio Technology (Beijing, China). Triplicate samples of all assays were constructed an independent library, followed by sequencing on an Illumina HiSeq sequencer (Illumina). Parameters for classifying significantly differentially expressed genes (DEGs) are |log2FC| ≥ 0.6 (FC: fold change of expressions) in the transcript abundance and q ≤ 0.05. KEGG pathway enrichment analysis was performed for the DEGs using Goseq R package and KOBAS 3.0 software (Available online: http://kobas.cbi.pku.edu.cn). KEGG pathway terms with *p*-value less than 0.05 were considered significantly enriched by target genes. RNA-seq data are accessible at NCBI (BioProject accession number: PRJNA608627).

### ELISA quantification of CCL3 and CCL4 production

MCL cells were treated with or without BTKi (2 μM) for 48 h in the presence of anti-human IgM F (ab)_2_ (#2022–01, Southern Biotech, Birmingham, AL), and CCL3 (#DMA00) and CCL4 (#DMB00) enzyme-linked immunoassay kits (R&D Systems, Minneapolis, MN) were used to determine CCL3 and CCL4 protein concentrations in the culture supernatants based on the manufacturer’s instructions.

### Stimulated Raman scattering (SRS) imaging in cultured cells

SRS imaging was performed on a femtosecond SRS microscope, with laser beating frequency tuned to the C-H stretching vibration band at 2845 cm^− 1^, as described previously [20]. No cell damage was observed during imaging procedure. LD area in the fields of view (*n* = 3) obtained from each sample was quantified using ImageJ. Specifically, “Threshold” function was used to select LDs in the cells due to their significantly higher signal intensities compared to other cellular structures. “Analyze Particles” function was then used to quantify the area fractions of LDs in the whole image area, then normalized to the cell number counted from the same image.

### Cell-viability assay

Cell viability was evaluated using AlamarBlue assay according to manufacturer’s protocol (Bio-Rad, Serotec). JeKo-1 and Mino cells were seeded in 96-well cell culture plates at 5 × 10^4^ cells/100 μl/well (*n* = 5). AlamarBlue solution (10 μl) was added to each well after cells were treated with BTKi at dose of 0, 0.25, 0.5, 1, 2 and 5 μM for 24, 48 and 72 h respectively. Fluorescence values were determined with a 560 nm excitation and 590 nm emission wavelengths after 3 h incubation in 37 °C. Cell-viability was calculated based on manufacturer’s instruction.

### Quantitative RT-PCR (qRT-PCR)

MCL cells were treated with or without BTKi (2 μM) for 48 h, and total RNA was extracted with TRIzol (#15596018; Thermo Fisher Scientific, Beijing) and RNA samples that meet following requirements were used in subsequent experiments: RNA integrity number (RIN) > 7.0 and a 28S:18S ratio > 1.8. To avoid genomic DNA contamination, total RNA samples were treated with a RNase-Free DNase Kit (Invitrogen) following manufacturer’s instructions and cDNA was synthesized from 2 μg of total RNA using Superscript III First-strand Synthesis System (#18080–051; Invitrogen, Beijing) according to manufacturer’s instructions. qRT-PCR was performed using PowerUp SYBR Green Mix (#00710493; Applied Biosystems, Beijing). The primers used for qRT-PCR were shown in Additional file 2: supplementary Table S1.

### Immunoblot analysis

MCL cells were treated with or without BTKi (2 μM) for 24 h, and cell lysates were prepared using Pierce™ RIPA buffer (#89900; Thermo Fisher Scientific) with Protease/Phosphatase Inhibitor Cocktail (#5872; Cell Signaling Technology) at 4 °C and quantified using Pierce™ BCA Protein Assay Kit (#23227; Thermo Fisher Scientific) before applying to a 10% polyacrylamide gel and transferring to a polyvinylidene difluoride membrane (#IPVH00010) from Immobilon-P. Antibodies against DGAT2 (#ab237613) and ENPP2 (#ab140915) were purchased from Abcam. Antibodies against SCD (#2794), ACACA (#3663), cleaved caspase-3 (#9661), cleaved PARP (#5625) and GAPDH (#5174) were all obtained from Cell Signaling Technology. Signals were detected using SuperSignal West Femto Maximum Sensitivity Substrate (#34095; Thermo Fisher Scientific) and images were acquired with Mini Chemiluminescent Imaging and Analysis System (Sage Creation Science, Beijing, China). Integrated optical density (IOD) of bands was evaluated by densitometry and analyzed using Gel-Pro Analyzer 4.0 software (Media Cybernetics, MD).

### Statistical analysis

Statistical analysis was performed with GraphPad Prism 8.0 (GraphPad Software Inc.). Data were shown as mean ± SEM. Differences between two groups or among multiple groups were determined by unpaired 2-tailed Student’s t-test or by one-way ANOVA with Tukey’s multiple comparisons test respectively. *P*-values less than 0.05 were considered significant.

## Results

### BTKi induced differential cytotoxicity against MCL cell lines

**Fig. 1:**
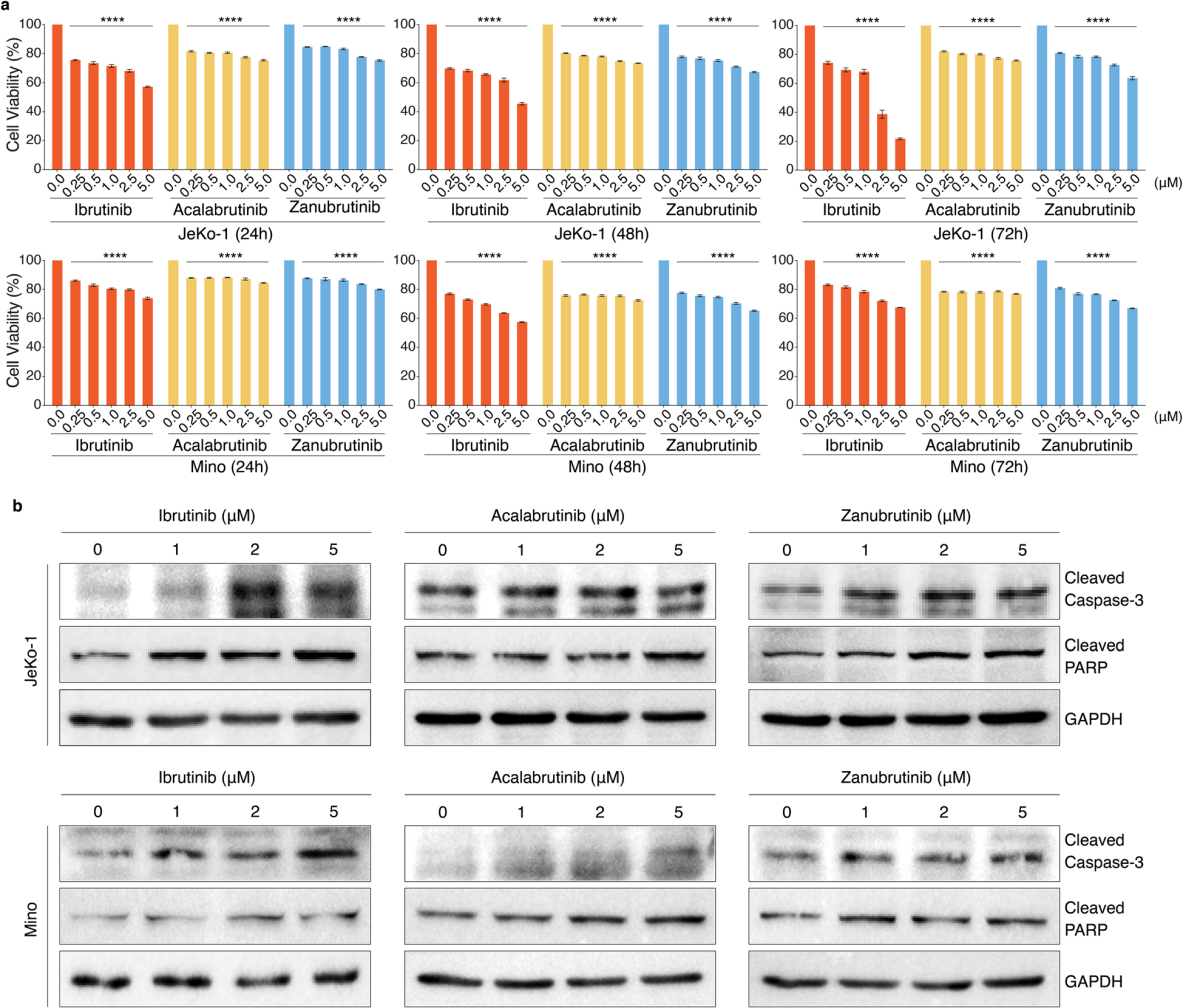
BTKi had differential apoptotic effects on MCL cells lines. **a** BTK inhibitors reduce MCL cell-viability. JeKo-1 and Mino cells were treated with Ibrutinib, Acalabrutinib or Zanubrutinib in a dose-dependent manner (0.25 μM, 0.5 μM, 1 μM, 2.5 μM, 5 μM) for 24, 48 and 72 h (*n* = 5). The cell-viability was determined by AlamarBlue assay. Data are shown as mean ± SEM; ****P < 0.0001, as calculated using the Student’s t-test. **b** Cleaved caspase-3 and cleaved PARP were increased in MCL cells, JeKo-1 and Mino, treated with Ibrutinib, Acalabrutinib or Zanubrutinib in a dose-dependent manner (1 μM, 2 μM, 5 μM) for 24 h, which were examined by immunoblot analysis. GAPDH was used as loading control

We firstly aimed to compare the cytotoxic effectiveness of Ibrutinib, Acalabrutinib and Zanubrutinib observed in MCL. The peak plasma concentration of Ibrutinib, Acalabrutinib and Zanubrutinib in patients treated with these drugs is about 0.5 μM [21, 22], 1.1 μM [16] and 1.4 μM [13] respectively, which can affect 100% occupancy and BTK inhibition. Accordingly, MCL cell lines, JeKo-1 and Mino, were treated with Ibrutinib, Acalabrutinib or Zanubrutinib individually at dose of 0, 0.25, 0.5, 1, 2 and 5 μM for 24, 48 and 72 h and measured cell-viability using AlamarBlue assay (Fig. 1a), which showed that Ibrutinib presented a stronger cytotoxic activity in JeKo-1 cells than in Mino cells, and the cytotoxicity of Acalabrutinib and Zanubrutinib were weak in both JeKo-1 and Mino cells over time. Such cytotoxic effects were accompanied by slight cleavage of caspase-3 and PARP (Fig. 1b and Additional file 1: Fig. S1), which were consistent with cytotoxic capacities of these three BTK inhibitors.

### RNA-seq identified apoptosis-related targets of BTKi

To understand the comprehensive mechanisms involved in MCL-cell apoptosis, we analyzed differentially expressed genes (DEGs) in Jeko-1 and Mino cells treated with Ibrutinib, Acalabrutinib or Zanubrutinib, versus untreated cells, via whole-transcriptome RNA sequencing (RNA-seq). The DEGs with |log2FC| ≥ 0.6 and q-value ≤0.05 were identified. A total of 2664 and 2587 DEGs were identified in JeKo-1 and Mino cells respectively. The overall hierarchical clustered graph showed that three repetitions of Ibrutinib, Acalabrutinib or Zanubrutinib treatment in JeKo-1 or Mino cells were clustered together, which mean the reproducibility were fairly good, and the differences between BTK inhibitor treatment were identified as well (Additional file 1: Fig. S2a), which might be caused by the different off-target effects of the three BTK inhibitors. The Venn diagram sets showed the number of DEGs regulated by Ibrutinib, Acalabrutinib or Zanubrutinib group in JeKo-1 or Mino cells (Additional file 1: Fig. S2b): 1832, 662 and 1891 DEGs were identified in JeKo-1 cells, and 1879, 1546 and 1399 DEGs were identified in Mino cells.

**Fig. 2:**
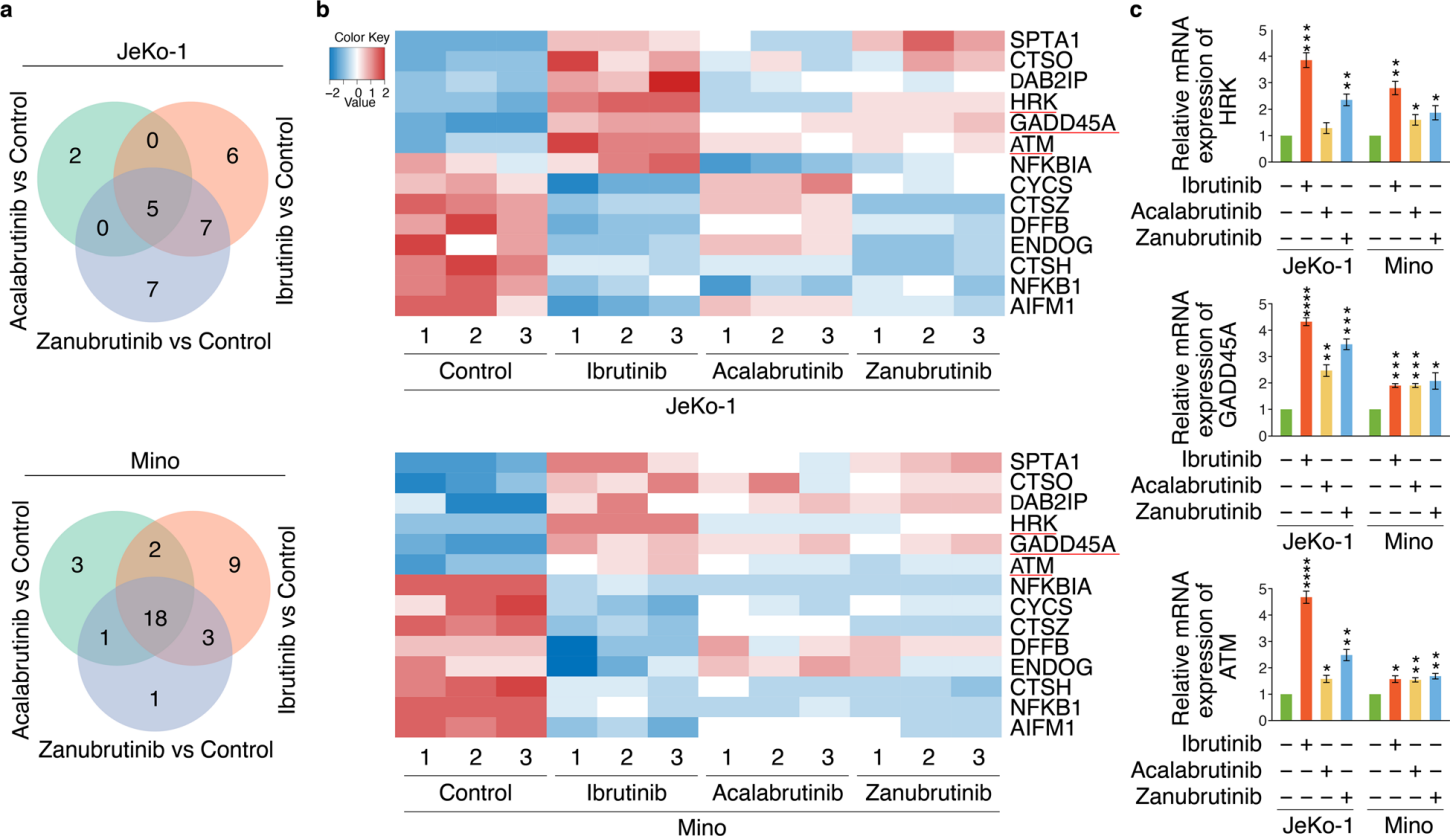
RNA-seq identified apoptosis-related targets of BTKi*.* **a** Venn diagram illustrating the number of genes involved in apoptosis which were commonly and differentially regulated by the treatment of Ibrutinib, Acalabrutinib or Zanubrutinib compared to control treatment in JeKo-1 and Mino cells. **b** Heatmap of apoptosis related DEGs regulated by Ibrutinib, Acalabrutinib or Zanubrutinib in both JeKo-1 and Mino cells, versus control respectively. Red represents upregulation and blue indicates downregulation. **c** Analysis of mRNA expression of *HRK*, *GADD45A* and *ATM* in JeKo-1 and Mino cells (*n* = 3) via qRT-PCR, which were treated with Ibrutinib, Acalabrutinib or Zanubrutinib (2 μM). Data are shown as mean ± SEM; *P < 0.05; **P < 0.01; ***P < 0.001; ****P < 0.0001, as calculated using one-way ANOVA with Tukey’s multiple comparisons test

KEGG enrichment analysis was used to perform a further functional classification and pathway assignment of DEGs regulated by Ibrutinib, Acalabrutinib or Zanubrutinib individually in JeKo-1 and Mino cells (Additional file 1: Fig. S3). Among KEGG enrichment pathways, DEGs involved in cell growth and death were of primary interest, from which apoptosis-related DEGs were screened. The numbers of these genes were shown in Venn diagram, which revealed relationships between each inhibitor in JeKo-1 and Mino groups (Fig. 2a). We next analyzed the overlapping set of apoptosis-related DEGs regulated by each BTK inhibitor in JeKo-1 or Mino respectively, followed by generating intersections of the DEGs in JeKo-1 and Mino (Fig. 2b), from which critical pro-apoptotic genes, *HRK*, *GADD45A* and *ATM*, were screened and validated by qRT-PCR. Obviously, Ibrutinib was more able to upregulate such apoptotic genes in JeKo-1 cells than in Mino cells, which might result in the more sensitivity of JeKo-1 cells to Ibrutinib than Mino cells. Acalabrutinib and Zanubrutinib had weak capabilities to change the expression of *HRK*, *GADD45A* and *ATM*, which may explain their low-level toxicities in both JeKo-1 and Mino cells (Fig. 2c). Such results revealed novel target genes regulated by these three BTK inhibitors, resulting in their distinct apoptosis-inducing abilities in MCL.

### RNA-seq identified chemotaxis-related target genes of BTKi

**Fig. 3:**
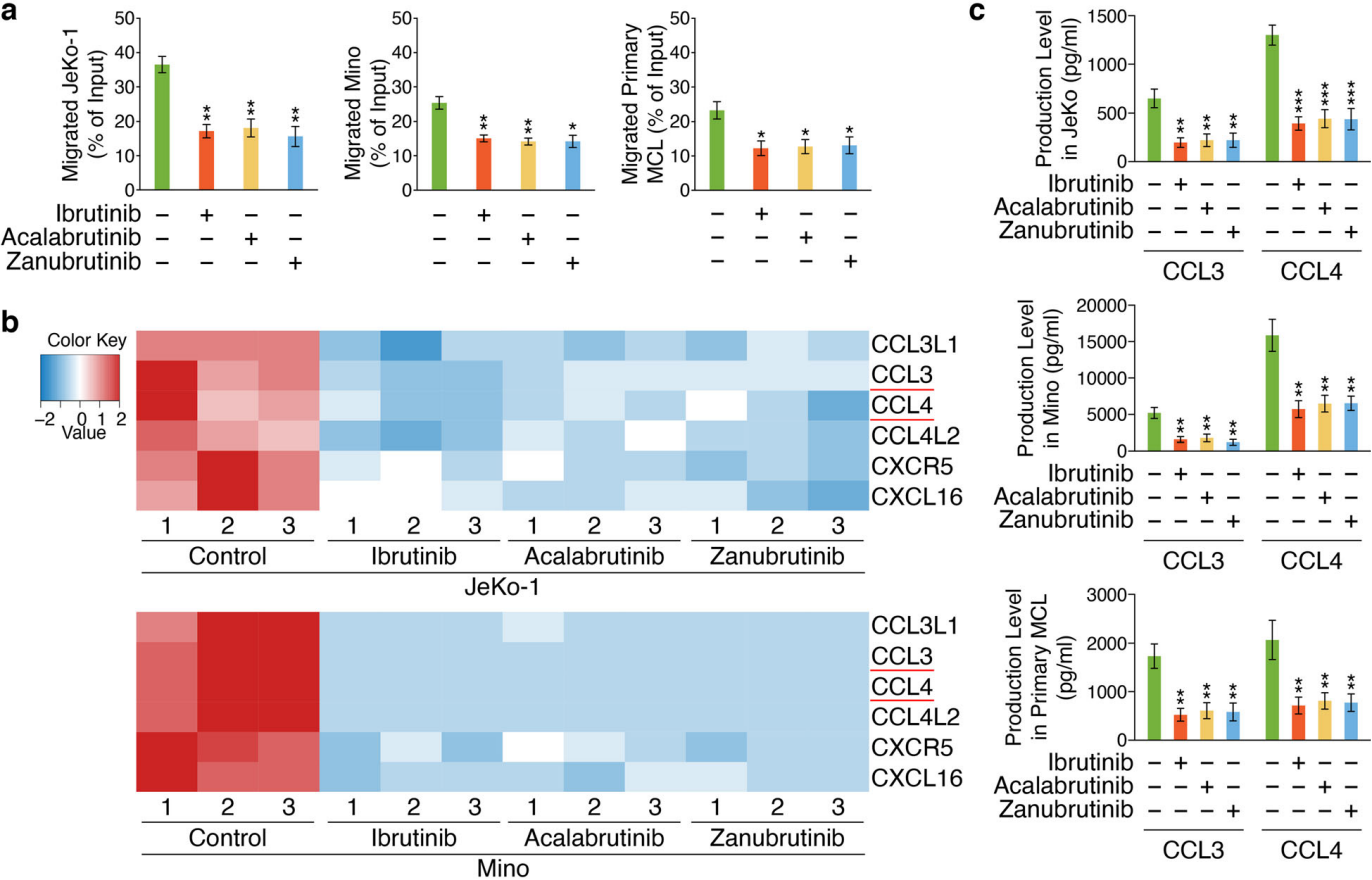
BTKi inhibit chemotactic migration in MCL cells. **a** Chemotaxis of JeKo-1, Mino and primary MCL cells (*n* = 3, primary MCL cells collected from patient 1–3.) was measured by a Transwell assay measuring migration towards either medium or stroma-conditioned medium for 8 h. Data are shown as mean ± SEM; *P < 0.05; **P < 0.01, as calculated using the Student’s t-test. **b** Heatmap of chemotaxis-related DEGs regulated by Ibrutinib, Acalabrutinib or Zanubrutinib in both JeKo-1 and Mino cells, versus control respectively. Red represents upregulation and blue indicates downregulation. **c** CCL3 and CCL4 levels were examined in the supernatants of JeKo-1, Mino and primary MCL cells (n = 3, primary MCL cells collected from patient 1–3.) treated with individual BTKi (2 μM) for 48 h compare to untreated cells in the presence of anti-human IgM F (ab)_2_. Data are shown as mean ± SEM; **P < 0.01; ***P < 0.001, as calculated using the Student’s t-test

Chemokines-activated BCR signaling facilitates cell migration (pseudoemperipolesis) beneath stromal cells [23]. In the current study, conditioned medium from HS-5 human marrow stromal cells induced migration of JeKo-1, Mino and primary MCL cells, which was inhibited by BTKi without significant differences between drug treatments at 2 μM (Fig. 3a). To understand the potentially distinct mechanisms underlying BTKi-controlled biological processes including MCL-cell migration, KEGG enrichment analysis was used to perform chemokine signaling pathway assignment of DEGs regulated by Ibrutinib, Acalabrutinib or Zanubrutinib individually in JeKo-1 and Mino cells, which was included in the immune system subcategory (Additional file 1: Fig. S3). We then analyzed the overlapping set of chemotaxis-related DEGs regulated by each BTKi in JeKo-1 or Mino respectively, followed by generating intersections of the DEGs in JeKo-1 and Mino, by which downregulated chemotaxis-related genes were screened, including *CCL3L1*, *CCL3*, *CCL4*, *CCL4L2*, *CXCL16* and *CXCR5* (Fig. 3b). Accordingly, we examined the levels of CCL3 and CCL4 in the supernatants of JeKo-1, Mino and primary MCL cells treated with or without BTKi individually for 48 h in the presence of anti-human IgM F (ab)_2_, and the results indicated that these three BTKi significantly suppressed the production of CCL3 and CCL4 with similar degree in MCL cells (Fig. 3c). These data suggested that the three BTKi demonstrated similar abilities to modulate chemotaxis-related genes, which may result in their similar MCL-cell chemotaxis inhibition.

### BTKi inhibited LD accumulation in MCL

**Fig. 4:**
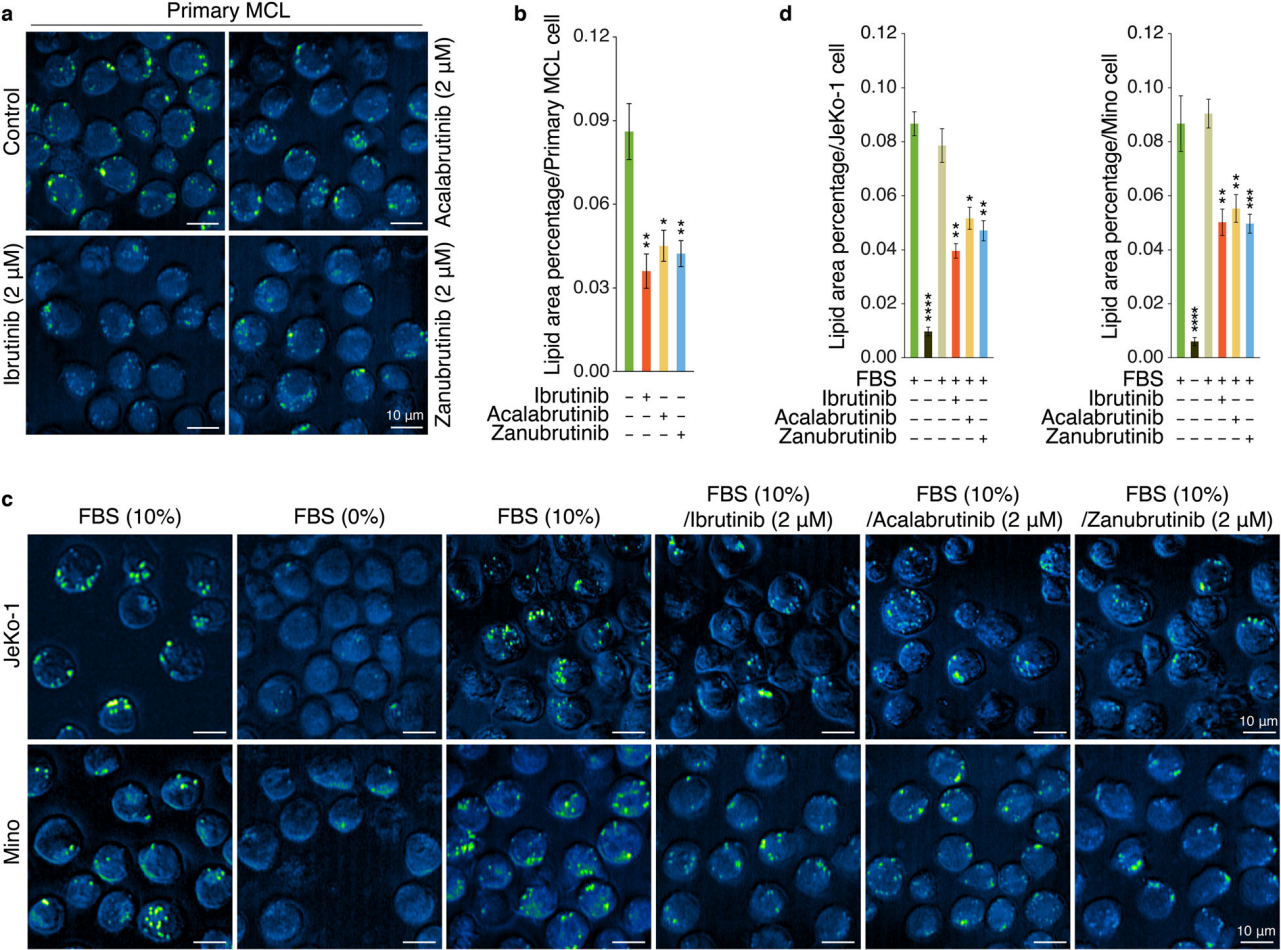
BTKi inhibited LD accumulation in MCL cells. **a** SRS imaging analysis of cellular LDs in primary MCL cells (n = 3, primary MCL cells collected from patient 1–3.) treated as the indicated conditions for 24 h. One representative sample is shown. Scale bars, 10 μm. **b** Lipid area in the fields of view (n = 3) obtained from each sample was quantified based on the SRS images from (**a**) by ImageJ software. Data are shown as mean ± SEM; *P < 0.05; **P < 0.01, as calculated using the Student’s t-test. **c** SRS imaging analysis of cellular LDs in JeKo-1 or Mino cells treated as the indicated conditions on the top of the figures. One representative MCL sample is shown. Scale bars, 10 μm. **d** Lipid area was quantified based on the SRS images from (**c**) (*n* = 5) by ImageJ software. Data are shown as mean ± SEM; *P < 0.05; **P < 0.01; ***P < 0.001; ****P < 0.0001, as calculated using the Student’s t-test

Considering the critical role of LDs for MCL-cell survival, we next aimed to investigate the capability of BTKi to block LD accumulation. Firstly, SRS imaging analysis was performed to test the LD accumulation in primary MCL cells treated with each BTKi for 24 h versus cells treated with vehicle control, which revealed that these three BTKi could significantly suppress LD accumulation (Fig. 4a, b). Next, JeKo-1 and Mino cells were treated based on the procedure shown in Additional file 1: Fig. S4, and analyzed by SRS imaging. The results showed that MCL cells cultured in 10% FBS media had abundant LDs, which diminished after serum-deprivation for 24 h unless provided with 10% FBS again, however, treatment with BTKi inhibited the capacity of FBS to stimulate the production of LDs significantly (Fig. 4c, d).

### RNA-seq identified LD accumulation related target genes of BTKi

**Fig. 5:**
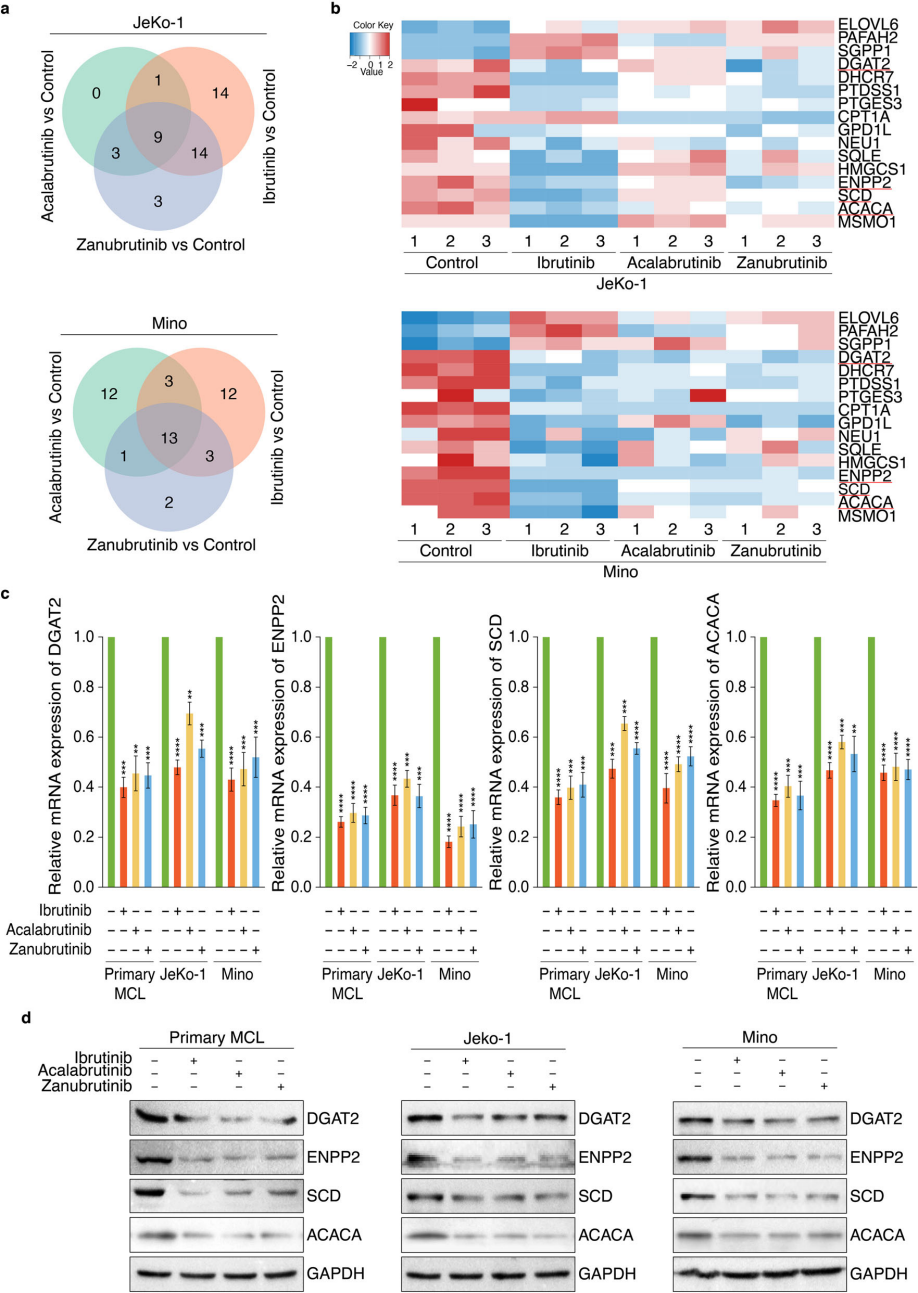
RNA-seq identified LD accumulation related targets of BTKi. **a** Venn diagram showing the relationship between DEGs of three drug treatment groups, which were involved in lipid metabolism. DEGs were analyzed by comparing Ibrutinib, Acalabrutinib or Zanubrutinib treatment group with untreatment control group. **b** Heatmap of cell growth and death related DEGs regulated by Ibrutinib, Acalabrutinib or Zanubrutinib in both JeKo-1 and Mino cells, versus control respectively. Red represents upregulation and blue indicates downregulation. **c** Analysis of mRNA expression of *DGAT2*, *ENPP2*, *SCD* and *ACACA* in MCL cells via qRT-PCR (n = 3), which were treated with Ibrutinib, Acalabrutinib or Zanubrutinib (2 μM), and primary MCL cells collected from patient 1–3. Data are shown as mean ± SEM; **P < 0.01; ***P < 0.001; ****P < 0.0001, as calculated using the Student’s t-test. **d** Analysis of protein expression of DGAT2, ENPP2, SCD and ACACA in MCL cells via immunoblot analysis, which were treated with Ibrutinib, Acalabrutinib or Zanubrutinib (2 μM), and primary MCL cells collected from patient 1

To reveal the molecular mechanism underlying BTKi-inhibited MCL-cell LD accumulation, KEGG enrichment analysis showed that BTKi could modulate the expression of some critical lipid metabolism-related DEGs (Additional file 1: Fig. S3), whose number was shown in Venn diagram (Fig. 5a). We then screened the overlapping set of lipid metabolism-related DEGs regulated by each BTKi in JeKo-1 or Mino respectively, followed by generating intersections of the DEGs in JeKo-1 and Mino (Fig. 5b), and four downregulated LD accumulation-related genes, *DGAT2*, *SCD*, *ACACA (ACC1)* and *ENPP2*, were selected and validated by qRT-PCR analysis (Fig. 5c) and immunoblot analysis (Fig. 5d and Additional file 1: Fig. S5). Since the inhibition of these four lipogenic genes could initiate tumor cell apoptosis [24–27], such findings may demonstrate novel mechanism underlying the anti-survival effects of these three BTKi in MCL via impairing lipid biosynthesis at least partially.

## Discussion

BTK is a major kinase in BCR signaling pathway, which is highlighted by the clinical effectiveness of irreversible small-molecule BTKi, Ibrutinib, Acalabrutinib and Zanubrutinib. In this study, we made a parallel analysis of Ibrutinib, Acalabrutinib and Zanubrutinib to uncover their potential similarities and differences in anti-survival effects in MCL, since they have differential binding selectivity against their common target, BTK, but have similar biologic effects and comparable clinical responses. Our MCL-cell viability data showed that Ibrutinib demonstrated a high cytotoxic activity in JeKo-1 cells, but not in Mino cells, which was consistent with previous findings that JeKo-1was classified as an Ibrutinib-sensitive cell line, while Mino was classified as an Ibrutinib-resistant cell line [28]. Also, the sensitivities of both JeKo-1 and Mino cells to Acalabrutinib and Zanubrutinib were low. In order to explain the mechanism underlying such effects, RNA-seq analysis followed by KEGG analysis were performed to identify the critical upregulated pro-apoptotic genes and downregulated anti-apoptotic genes controlled by each BTKi, and the data showed that Ibrutinib was more powerful to upregulate pro-apoptotic genes, *HRK*, *GADD45A* and *ATM* in JeKo-1 cells than in Mino cells. In addition, Acalabrutinib and Zanubrutinib had low capacities to modulate the expression of such three apoptotic genes in both JeKo-1 and Mino cells. Of note, these three apoptosis-related genes identified in this study were well-known in apoptosis signaling. GADD45A is associated with DNA damage and is proapoptotic [29], and ATM, as a tumor suppressor gene, plays a role in the initiation and/or progression of MCL [30]. As a member of the pro-apoptotic subgroup of BCL-2 family, HRK is an essential initiators of apoptosis that can function as tumor suppressors [31]. All of these findings could support the idea that these genes play roles in mediating MCL-cell apoptosis induced by BTK inhibitors, even though overall apoptotic effects induced by BTKi were moderate in MCL.

Both Ibrutinib and Acalabrutinib have been shown to decrease levels of CCL3 and CCL4, two critical chemokines inducing migration or homing of leukemia cells, in CLL-cell cultures and their separate clinical trials [14–16, 32]. Zanubrutinib could inhibit homing of CLL cells through downregulating CXCR5, a homing receptor mediating migration or homing and BCR signaling activation [18, 33]. However, regulatory impact of these three BTKi on chemotaxis and chemotaxis-related genes in MCL still need to be analyzed. Our study showed that conditioned medium from HS-5 human marrow stromal cells induced migration of MCL cells, which was inhibited by Ibrutinib, Acalabrutinib and Zanubrutinib without significant differences between drug treatments. Consistent with these functional data, our results indicated that Ibrutinib, Acalabrutinib and Zanubrutinib similarly reduced the expression of *CCL3*, *CCL4* and *CXCR5* via RNA-seq followed by KEGG analysis, and the production of CCL3 and CCL4 was validated by ELISA quantification, which did not show significant differences between drug treatments as well. Except these known chemotaxis-related target genes of BTKi, we also found that Ibrutinib, Acalabrutinib and Zanubrutinib could similarly reduce the expression of *CCL3L1*, *CCL4L2* and *CXCL16*.

Since elevated LDs could enhance MCL-cell survival [34], we detected whether these three BTKi could inhibit pro-survival LD accumulation in MCL-cell via SRS imaging analysis, a label-free live-cell imaging technique for testing intracellular components accumulation, including LDs [35]. As expected, quantitative analysis of lipogenesis at single-cell level via SRS imaging revealed that treatment with BTKi significantly reduced the accumulation of LDs in MCL. Based on the KEGG classification of RNA-seq data, we found that BTKi treatment dramatically reduced the expression of several pivotal lipogenic genes, *DGAT2*, *ENPP2*, *SCD* and *ACACA* (*ACC1*). DGAT2 catalyzes the final step in synthesis triglyceride, which is a major component of LDs [36], and genetic deletion of DGAT2 was lethal with knockout mice presenting severe and systemic reductions in triglyceride [37]. SCD is a principal enzyme responsible for fatty acid desaturation, which is critical for growth, survival and tumorigenesis [25, 38]. ENPP2, also known as Autotaxin (ATX), is always overexpressed in many malignancies [39], including MCL [40]. Interestingly, ATX catalyzes the extracellular biosynthesis of lysophosphatidic acid (LPA), and LPA is responsible for cancer cells growth and anti-cancer therapy resistance of many cancer cells [27]. Combined with our findings showing BTKi-associated ENPP2 downregulation, the downregulation of SCD might be caused by the reduction of LPA levels at least partially, since LPA could stimulate SCD expression and therefore accelerate the formation of lipid droplets [41]. Besides, ACC1, also known as ACACA, controls de novo lipogenesis, whose chemical inhibition suppresses lipogenesis and induces apoptosis in cancer cells [26]. Previous study showed that Zanubrutinib could downregulate the expression of ACACA in MCL [19], and our study demonstrated that both Ibrutinib and Acalabrutinib downmodulated ACACA expression in MCL as well. Importantly, inhibition of fatty acid synthesis, a crucial step of LDs accumulation, triggers significant apoptosis in MCL [17]. Accordingly, our data suggested that BTKi-induced downregulation of *DGAT2*, *ENPP2*, *SCD* and *ACACA* might result in LD accumulation inhibition, which trigger modest MCL-cell death at least partially, and such findings provide a new evidence that targeting the lipid metabolism pathway might be a strategy to treat MCL, or other B-cell malignancies, which deserves further studies.

## Conclusions

BTKi differentially initiate MCL-cell apoptosis via modulating distinct apoptotic target genes, and similarly inhibit MCL-cell chemotactic homing or LD accumulation due to their similar capabilities to regulate chemotaxis or LD accumulation related target genes.

## Supplementary Information

**Additional file 1.**

**Additional file 2.**

## Data Availability

All data generated or analyzed during this study are included in this published article and its supplementary information files. RNA-seq data are accessible at NCBI (BioProject accession number: PRJNA608627).

## Abbreviations

MCL: Mantle cell lymphoma
BTK: Bruton’s tyrosine kinase
BCR: B cell receptor
SRS: Stimulated Raman scattering
LD: Lipid droplet
DEGs: Differentially expressed genes
RNA-seq: RNA sequencing
KEGG: Kyoto Encyclopedia of Genes and Genomes
HRK: Harakiri
GADD45A: Growth Arrest And DNA Damage Inducible Alpha
ATM: Ataxia-Telangiesctasia mutated Serine/Threonine Kinase
CCL3: Chemokine (C-C motif) ligand 3
CCL4: Chemokine (C-C motif) ligand 4
CXCR5: C-X-C chemokine receptor type 5
CCL3L1: Chemokine (C-C motif) ligand 3-like 1
CCL4L2: Chemokine (C-C motif) ligand 4-like 2
CXCL16: Chemokine (C-X-C motif) ligand 16
DGAT2: Diacylglycerol O-Acyltransferase 2
ENPP2: Ectonucleotide Pyrophosphatase/Phosphodiesterase 2
SCD: Stearoyl-CoA desaturase
ACACA/ACC1: Acetyl-CoA Carboxylase Alpha
LPA: Lysophosphatidic Acid
LPL: Lipoprotein Lipase
FASN: Fatty Acid Synthase
