# Supplementary material for: Distinct BTK inhibitors differentially induce apoptosis but similarly suppress chemotaxis and lipid accumulation in mantle cell lymphoma
Source: BMC Cancer. 2021 Jun 26;21:732. doi: 10.1186/s12885-021-08475-3 (PMC8235860; doi:10.1186/s12885-021-08475-3)
Supplement: Supplementary file 1 — Additional file 1. [file 12885_2021_8475_MOESM1_ESM.pdf]

Additional file 1: Supplementary Appendix

Supplement to: Zhuojun Liu,et al., Distinct BTK inhibitors differentially induce apoptosis but similarly suppress chemotaxis and lipid accumulation in mantle cell lymphoma.

Figure S1

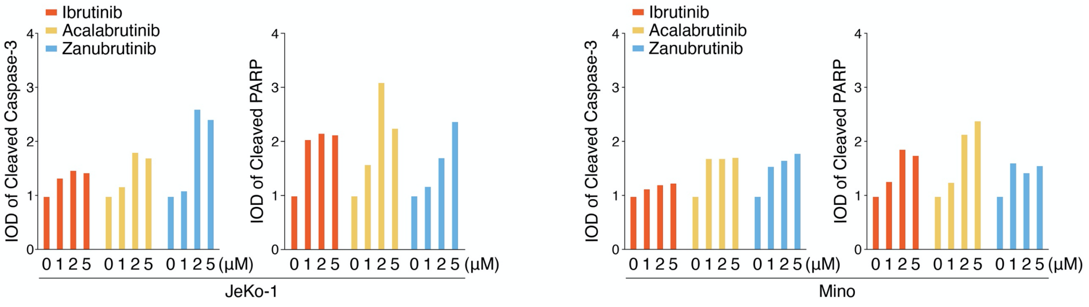

Supplementary Figure S1. Integral optical density (IOD) values of Figure 1b. Each bar represents the ratio of band IOD values for cleaved caspase-3 and cleaved PARP versus GAPDH normalized with respect to untreated samples in JeKo-1 and Mino cells.

Figure S2

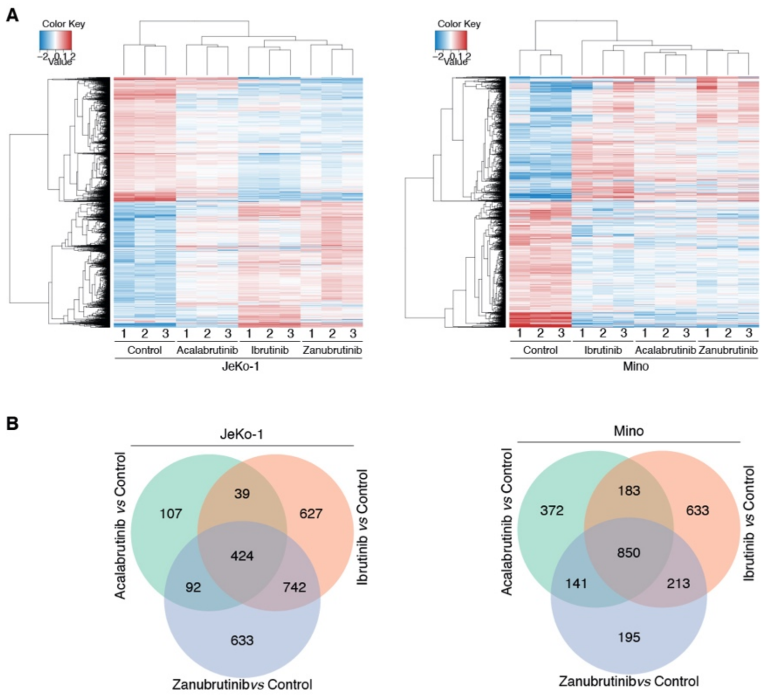

Supplementary Figure S2. RNA-seq analysis of BTKi targets. **(A)** Heatmap of DEGs regulated by Ibrutinib, Acalabrutinib and Zanubrutinib versus control in JeKo-1 and Mino cells. **(B)** Venn diagram illustrating the number of DEGs regulated by Ibrutinib, Acalabrutinib or Zanubrutinib compared to control in JeKo-1 and Mino cells.

Figure S3

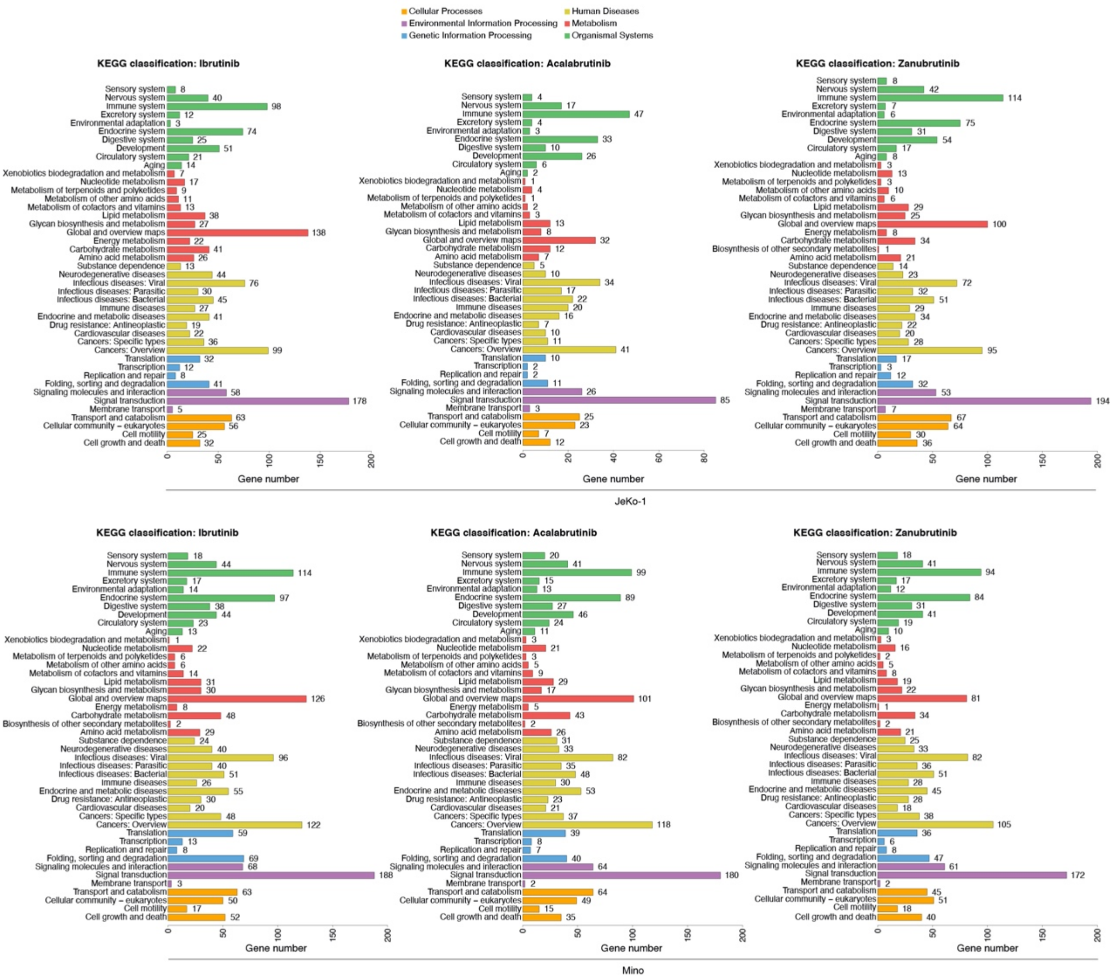

Supplementary Figure S3. KEGG analysis of altered pathways induced by BTKis. Functional classification of KEGG pathway was analyzed by using the DEGs regulated by Ibrutinib, Acalabrutinib or Zanubrutinib in JeKo-1 or Mino cells. The bar length represents the observed number of genes within the respective KEGG pathway. The y-axis indicates the name of the KEGG pathways. The x-axis represents the observed number of genes annotated under that pathway.

Figure S4

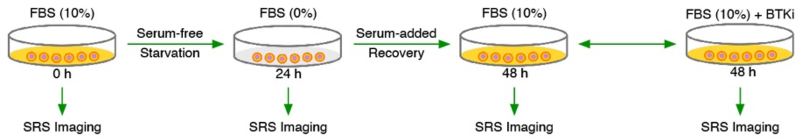

Supplementary Figure S4. MCL-cell sample preparation flowchart for SRS imaging analysis of lipid metabolism. The procedure of cell starvation, FBS and/or BTKi treatment were indicated.

Figure S5

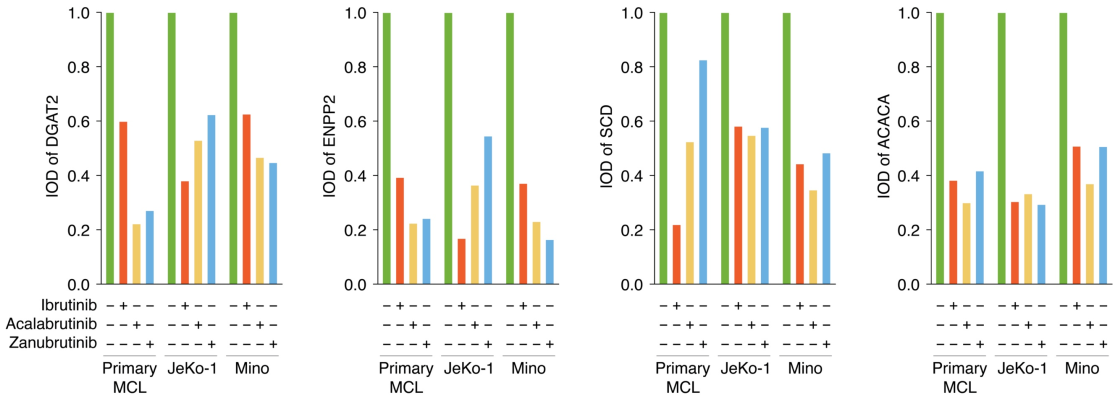

Supplementary Figure S5. IOD values of Figure 5d. Each bar represents the ratio of band IOD values for DGAT2, ENPP2, SCD and ACACA versus GAPDH normalized with respect to untreated samples in primary MCL, JeKo-1 and Mino cells.
