# Supplementary material for: Distinct BTK inhibitors differentially induce apoptosis but similarly suppress chemotaxis and lipid accumulation in mantle cell lymphoma
Source: BMC Cancer. 2021 Jun 26;21:732. doi: 10.1186/s12885-021-08475-3 (PMC8235860; doi:10.1186/s12885-021-08475-3)
Supplement: Supplementary file 2 — Additional file 2. [file 12885_2021_8475_MOESM2_ESM.pdf]

Additional file 2: Supplementary Appendix

Supplement to: Zhuojun Liu,et al., Distinct BTK inhibitors differentially induce apoptosis but similarly suppress chemotaxis and lipid accumulation in mantle cell lymphoma.

Supplementary Table 1. The primers used for qRT-PCR validation.

Gene name	Primer sequence	
GADD45A	Forward	5'-CTGGAGGAAGTGCTCAGCAAAG
	Reverse	5'-AGAGCCACATCTCTGTCGTCGT
ATM	Forward	5'-TGTTCCAGGACACGAAGGGAGA
	Reverse	5'-CAGGGTTCTCAGCACTATGGGA
HRK	Forward	5'-CCACAGCACTGCTAGTTCCA
	Reverse	5’-TCCCCGGGGCCTTATAGAAA
DGAT2	Forward	5'-GCTACAGGTCATCTCAGTGCTC
	Reverse	5'-GTGAAGTAGAGCACAGCGATGAG
ENPP2	Forward	5'-TATGCTGCGGAAACTCGTCAGG
	Reverse	5'-GACGTTGACACACCGATGCAGT
SCD	Forward	5'-CCTGGTTTCACTTGGAGCTGTG
	Reverse	5'-GTGGTGAAGTTGATGTGCCAGC
ACACA	Forward	5'-TTCACTCCACCTTGTCAGCGGA
	Reverse	5'-GTCAGAGAAGCAGCCCATCACT
GAPDH	Forward	5'-GTCTCCTCTGACTTCAACAGCG
	Reverse	5'-ACCACCCTGTTGCTGTAGCCAA
